# Interstitial Pregnancy: Case Report of Atypical Ectopic Pregnancy

**DOI:** 10.7759/cureus.8081

**Published:** 2020-05-13

**Authors:** Lucas Tadeu Rocha Santos, Sebastião Carlos de Sousa Oliveira, Liana Gonçalves Aragão Rocha, Nathaniel dos Santos Sousa, Rosenildo de Sousa Figueiredo

**Affiliations:** 1 Internal Medicine, Federal University of Ceara, Sobral, BRA; 2 Neurological Surgery, Federal University of Ceara, Sobral, BRA; 3 Obstetrics and Gynecology, Santa Casa de Misericórdia Hospital, Sobral, BRA; 4 Obstetrics and Gynecology, Sao Camilo Hospital, Tiangua, BRA

**Keywords:** interstitial pregnancy, ectopic pregnancy, case report

## Abstract

Interstitial or cornual implantation of the blastocyst is rare, accounting for 2% to 3% of ectopic pregnancies, being considered not viable. The important complications of interstitial pregnancy are uterine rupture and massive bleeding, which usually occur before 12 weeks of pregnancy. The authors report a case of a 36-year-old woman with complaints of transvaginal bleeding and abdominal pain associated with amenorrhea for seven weeks and positive beta-human chorionic gonadotropin (HCG). Transvaginal ultrasound and exploratory laparotomy were performed, confirming the diagnosis of interstitial ectopic pregnancy. The patient underwent a salpingectomy and cornual resection on the left, evolving with clinical improvement and hospital discharge.

## Introduction

Ectopic pregnancy (EP) occurs when the blastocyst is implanted outside the uterine endometrium, often in the fallopian tube, and represents 2% of pregnancies [1]. The incidence of EP is difficult to determine. Among women who come to the emergency department with complaints of transvaginal bleeding in the first trimester and/or pain, the incidence ranges from 6% to 16%, and African American patients have higher mortality rates [2]. 

The main risk factors for EP are a previous history of EP and tubal surgery. However, half of the cases do not have risk factors for the development of the condition. Other risk factors are pelvic inflammatory disease, infertility, current or previous use of an intrauterine device, exposure to diethylstilbestrol, age over 40 years, smoking and assisted reproduction [1].

The main site of blastocyst implantation in EP occurs in the fallopian tubes. Other implantation forms are cervical, ovarian, abdominal, in the cesarean section scar and intramural. In the tuba, the most common sites are ampulla (70%), isthmus (12%), fimbriae (11%) and interstitial (2.4%) [3]. The interstitial portion of the uterine tube represents the proximal segment incorporated into the uterine musculature.

## Case presentation

**Figure 1:**
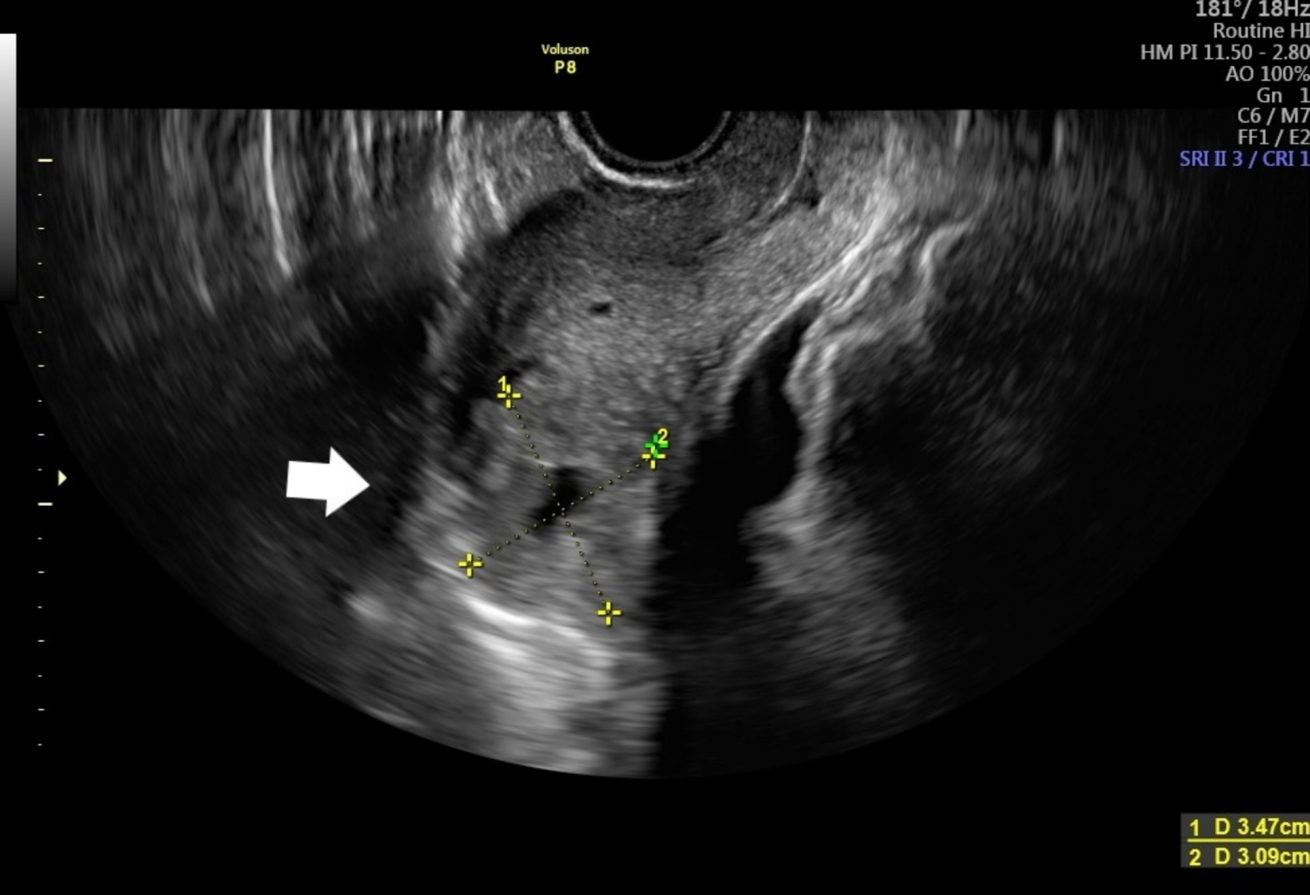
Transvaginal ultrasound Transvaginal ultrasound indicating echogenic structure in left cornual region (arrow).

**Figure 2:**
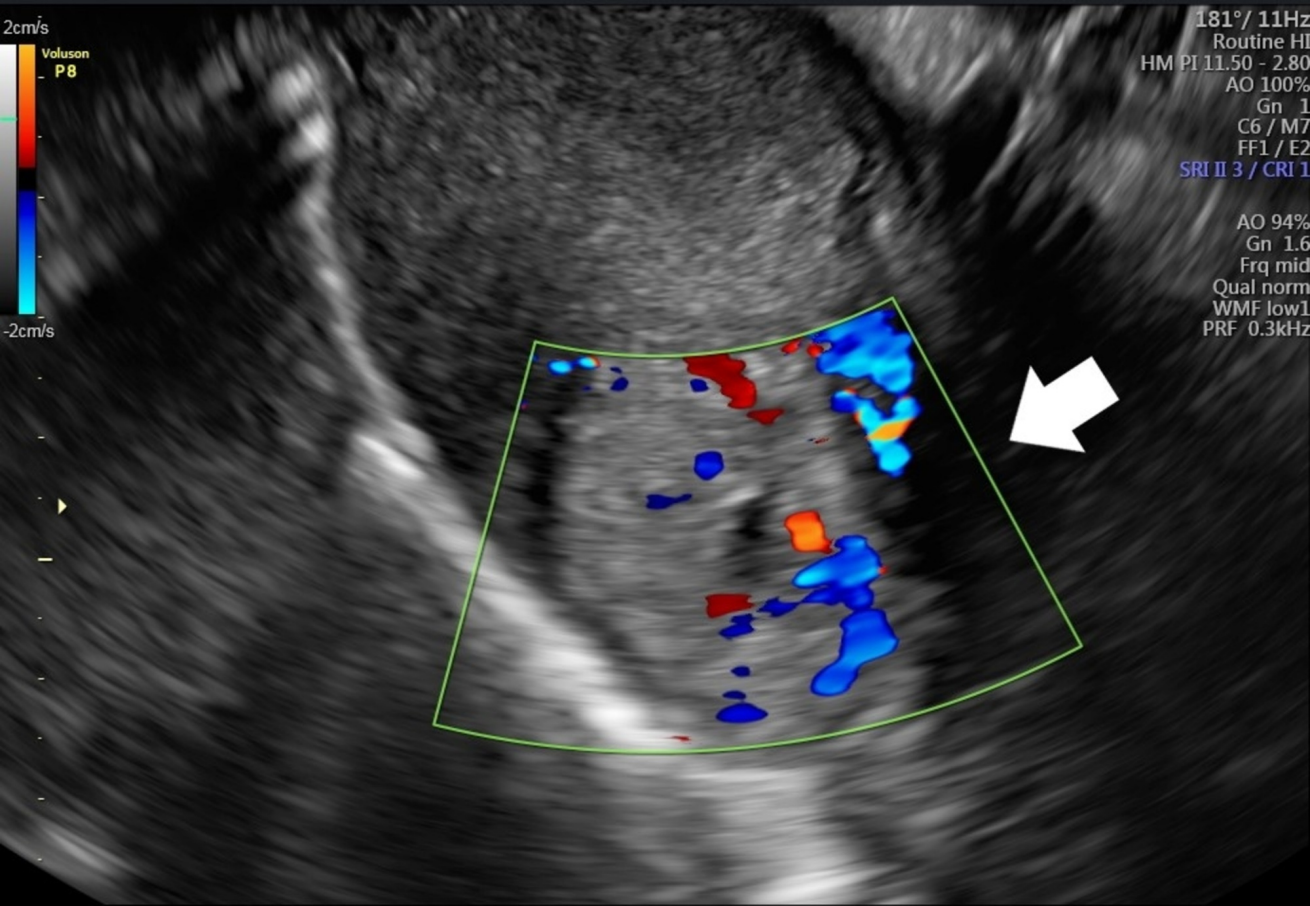
Doppler transvaginal ultrasound Doppler transvaginal ultrasound indicating echogenic structure in left cornual region (arrow).

A 36-year-old woman, G2P0A1, sought medical attention at the hospital’s maternity due to transvaginal bleeding with clots and abdominal pain, located on hypogastric region; the pain was of intermittent frequency and was characterized as colic pain, with an intensity of 6 out of 10 on the numerical pain scale. The patient reported that the pain improved with rest and was aggravated by movement and that it had started 20 days ago, presenting progressive clinical improvement on the previous days. In addition, the patient reported amenorrhea seven weeks ago and had a positive qualitative serum beta-human chorionic gonadotropin (HCG) test. Upon admission, the patient was clinically and hemodynamically stable and without signs of peritonism. Specular examination was performed, and active bleeding through the external orifice of the cervix was absent. In her personal and pathological history, she mentioned an abortion during the first trimester of pregnancy that occurred six months before admission. In that occasion, uterine curettage was performed. She denied ectopic or molar pregnancy, pelvic inflammatory disease, genital infections, vulvovaginitis, tubal reconstruction or previous in vitro fertilization (IVF). The patient was admitted to the hospital, and transvaginal ultrasound was performed showing a uterus in anteversion and anteflexion with an endometrium measuring 10 mm and a mass in the myometrial region located at the left cornual region, measuring 3.47 cm in the largest diameter, with degenerated gestational sac inside, without a fetal heartbeat. The diagnosis of interstitial EP was hypothesized (Figures 1, 2).

**Figure 3:**
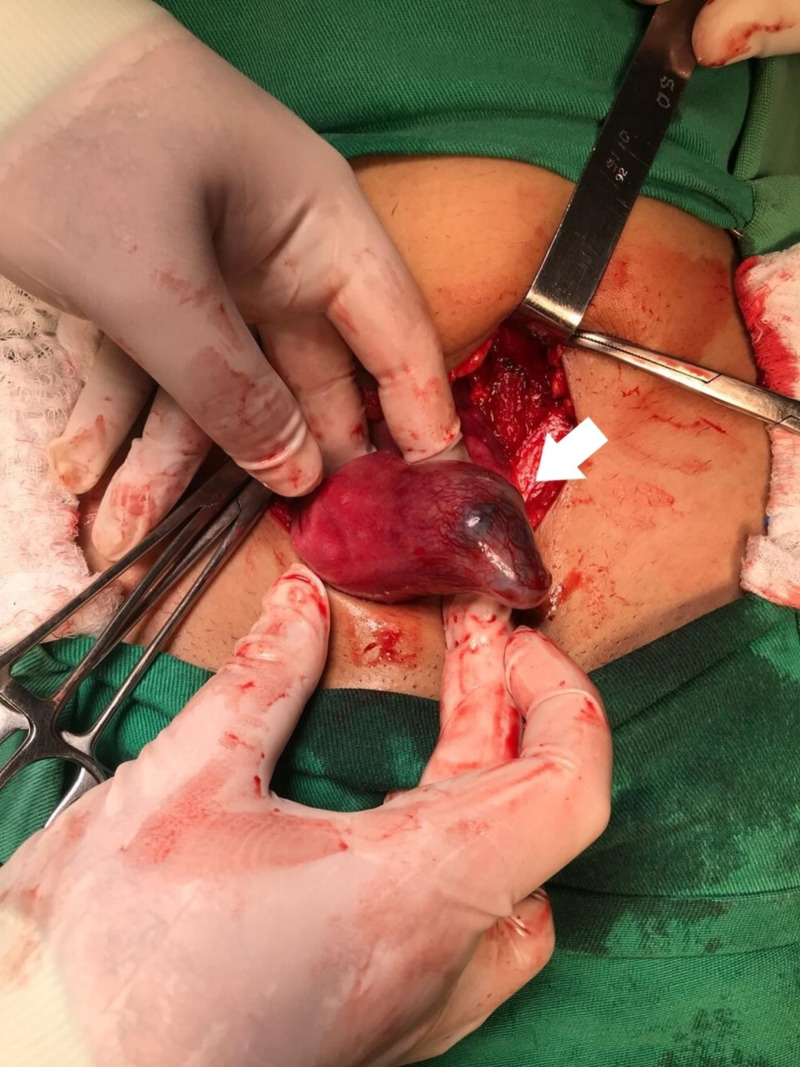
Transoperative image Ectopic interstitial pregnancy not ruptured.

The case was discussed with the patient and the staff opted for resolution of the condition through exploratory laparotomy. During the surgical procedure, an intact interstitial EP was confirmed (Figure 3), and a left salpingectomy was performed associated with resection of the interstitial portion of the left uterine tube. The patient evolved without surgical complications and was discharged from the hospital following standard laparotomy protocol. 

## Discussion

Despite being used as a synonym, interstitial and cornual pregnancy represents two distinct conditions. Originally, cornual implantation described implantation in the lateral and upper portion of the uterine cavity, in a horn of a bicornuate uterus, in a rudimentary horn of a unicorn uterus or on the side of a septate uterus. The interstitial denomination, on the other hand, refers to implantation in the intramural proximal portion of the tube [4].

The classic triad of EP consists of amenorrhea followed by transvaginal bleeding in the first trimester and abdominal or pelvic pain. However, in some cases, patients may present no symptoms or as a surgical emergency [5].

The diagnosis of EP requires the confirmation of pregnancy and its ectopic location associated with the assessment of the patient's hemodynamic stability.

Transvaginal ultrasonography is an imaging exam that is very useful for locating pregnancy through the localization of an intrauterine gestational sac, an embryo or an ectopic gestational sac, with or without a fetal heartbeat. The ultrasound signals used to diagnose interstitial pregnancy are the eccentric location of the gestational sac proximal to the uterine horn, the presence of an interstitial line (defined as an echogenic line that extends from the most superior and lateral aspect of the endometrium to the middle portion of the mass or interstitial sac), failure to see the myometrium completely surrounding the sac and failure of the gestational sac to communicate with the endometrium [6]. The interstitial line probably represents the endometrial canal or the interstitial portion of the uterine tube, depending on the size of the pregnancy. In cases of rupture, the typical finding is blood in the peritoneal cavity [7].

Differential diagnoses for EP are other causes of pain and bleeding in the first trimester of pregnancy, such as spontaneous abortion, gestational trophoblastic disease, subchorionic hematoma and pathologies of the vagina, cervix and uterus. The presence of an endometrial layer measuring less than 5 mm associated with a gestational sac separated from the endometrium is indicative of interstitial pregnancy, but has low specificity. The presence of the interstitial line has a sensitivity of 100% and a specificity of 80% for interstitial EP [8].

Tubal EP can be treated expectantly, clinically (with methotrexate) or surgically. The criteria for performing treatment with methotrexate are hemodynamic stability, absence of fetal cardiac activity at ultrasound examination and pre-treatment beta-HCG below 5,000 mIU/mL. Surgical intervention is necessary in women with hemodynamic instability, suspected or confirmed tubal rupture, heterotopic pregnancy and contraindication or failure to treat with methotrexate [1].

When the diagnosis of interstitial pregnancy was late and occurred due to rupture, conservative clinical and surgical treatment was not possible, with hysterectomy and cornual resection being performed in most cases. However, advances in the diagnosis techniques allow the removal of the interstitial pregnancy by laparoscopic cornuostomy with cornual resection, when the surgical procedure is necessary [9].

After resolution, patients with interstitial pregnancies should be informed about the increased risk of EP and consequent uterine rupture. Due to that fact, strict prenatal care is needed in a new pregnancy, with an indication of elective cesarean delivery at term in order to avoid the risk of uterine rupture during labor [10] .

In the case described previously, a case of interstitial EP with a clinical condition similar to the clinical presentation described in the literature (occurring between six and eight weeks after the last menstrual period) is presented [11]. The occurrence of tubal rupture varies between studies, reaching 20% to 50% of cases and occurring before 12 weeks of gestation [12]. Mortality from interstitial EP is 2% to 2.5% and accounts for 20% of deaths from EP [13]. The diagnosis of EP in the case was carried out by means of proof of pregnancy by beta-HCG, ultrasound characteristics of cornual EP and by laparotomy. This surgical technique was chosen due to the unavailability of a diagnostic laparoscopy procedure at the hospital.

## Conclusions

Ectopic interstitial pregnancy is a rare presentation of EP. However, the disease can develop with hemodynamic instability and death. Thus, it is important to promptly recognize the classic clinical presentation, as well as ways to perform its diagnosis, differential diagnoses and clinical and surgical management. In addition, patients should be advised of the risks associated with new pregnancies.
